# Sex Modified the Association between Sleep Duration and worse Cognitive Performance in Chinese Hypertensive Population: Insight from the China H-Type Hypertension Registry Study

**DOI:** 10.1155/2022/7566033

**Published:** 2022-06-24

**Authors:** Xinlei Zhou, Junpei Li, Chao Yu, Wangsheng Fang, Yanyou Xie, Li Wang, Si Shen, Wei Zhou, Lingjuan Zhu, Tao Wang, Xiao Huang, Huihui Bao, Jianglong Tu, Xiaoshu Cheng

**Affiliations:** ^1^Department of Cardiovascular, The Second Affiliated Hospital of Nanchang University, Nanchang Jiangxi, China; ^2^Jiangxi Provincial Cardiovascular Disease Clinical Medical Research Center, Nanchang Jiangxi, China; ^3^Jiangxi Sub-Center of National Clinical Research Center for Cardiovascular Diseases, China; ^4^Center for Prevention and Treatment of Cardiovascular Diseases, The Second Affiliated Hospital of Nanchang University, Nanchang, China; ^5^Wuyuan Health Commission, Wuyuan, China; ^6^Department of Neurology, The Second Affiliated Hospital of Nanchang University, Nanchang, China

## Abstract

**Objectives:**

Cognitive decline could be seen as the sign of preclinical phase of dementia, which was found to be sex differentiated. Previous studies had discovered that there might be some link between abnormal sleep duration and cognitive performance. Additionally, hypertension was found to be one of the important risk factors for cognitive decline and abnormal sleep duration was also a significant risk factor for hypertension. Therefore, the purpose of this study was to investigate sex differences in the association of sleep duration with cognitive performance and to further explore potential effect modifiers that may exist.

**Methods:**

Data analyzed in this study was from the China H-type Hypertension Registry Study. Sleep duration was assessed with a sleep questionnaire and categorized as <5 hours, 5-8 hours, and ≥8 hours. Cognitive performance was evaluated with the Mini-Mental State Examination (MMSE).

**Result:**

A total of 9527 subjects were included. The average age was 63.7 ± 9.8 years. Linear regression analyses showed that the association between long sleep duration (≥8 h) and MMSE score adjusting for pertinent covariables was stronger in female (*β* = −0.95, 95% CI: -1.23 to -0.68, *P* < 0.001) than in male (*β* = −0.29, 95% CI: -0.53 to -0.06, *P* = 0.013). Furthermore, there was a significant interaction between sleep duration and age on cognitive performance only in female.

**Conclusion:**

In summary, this study found that long sleep duration (≥8 h) was associated with poorer cognitive performance. Furthermore, this association was more pronounced in female than in male, especially in older female.

## 1. Introduction

As the main cause of disability of the elderly, dementia affects the most populous country globally that patients living with dementia in China account for 25% of the global cases [1]. It has been estimated that 46.8 million people suffered from dementia worldwide in 2015, and this number will grow to 131.5 million by 2050 [2]. It is considered a global public health challenge that the treatment and care of dementia place heavy burdens on social, medical, and economic realities. However, there are currently fewer drugs available to treat dementia, and no effective drugs were found which can significantly delay the progression of dementia. Cognitive decline could be seen as the sign of preclinical phase of dementia [3]. Additionally, hypertension was found to be one of the important risk factors for cognitive decline [4]. According to the recent Chinese hypertension survey, the hypertensive patients in 2018 accounted for approximately 27.9% among adults over 18 years old [5]. Currently, China has entered an aging society [6] and hypertension prevalence increases with aging [5, 7]. Therefore, exploring other modifiable risk factors was of great clinical value for the early prevention of cognitive decline, especially in adults with hypertension.

Sleep was critical for organismal health, and previous studies had discovered that there might be some link between abnormal sleep duration and cognitive performance. Xu et al. [8] confirmed that both excess sleep and insufficient sleep duration could cause amyloid deposition at the biomarkers level for the first time. It is well known that amyloid deposition could exacerbated cognitive impairment [9]. Indeed, abnormal sleep duration was also a significant risk factor for hypertension [10]. Therefore, it was necessary to investigate the association between sleep duration and cognitive performance in adults with hypertension. However, evidence on the association in adults with hypertension is limited.

Previous studies have found that sex differences might exist in cognitive performance and cognitive decline. Li et al. [11] not only found that females presented better episodic memory ability than males but also that they argued that cognitive function in males is better than in female. Furthermore, previous studies reported that females showed significantly faster age-related decline and greater cognitive deterioration than elderly males [12–14]. Therefore, the impact of sex on the association between sleep duration and cognitive function was worthy of deserved further exploration.

The purpose of this study was to investigate sex differences in the association of sleep duration with cognitive performance and to further explore potential effect modifiers that may exist.

## 2. Methods

### 2.1. Study Design and Participants

Data analyzed in this study were from the China H-type Hypertension Registry Study (Registration number: ChiCTR1800017274). Previous literature described in detail the exclusion criteria and the method of data collection [15, 16]. This was an observational study conducted in Wuyuan, China, from March 2018. Adults (aged 18 years or over) who experienced hypertension were the subjects of this study. Hypertension was defined as systolic blood pressure (SBP) ≥ 140 mmHg or/and diastolic blood pressure (DBP) ≥ 90 mmHg when blood pressure was measured in a resting and sitting position or taking antihypertensive medications or self-report of a hypertensive diagnosis. Exclusion criteria were described as follows: (1) mental or neurological abnormalities that prevent cooperation with the investigation, (2) unable to complete follow-up due to poor adherence or planned to relocate in recent, and (3) the participants assessed by the study physicians as unsuitable for inclusion or long-term follow-up.

A total of 14268 individuals were enrolled in the China H-type Hypertension Registry Study. We included nonhypertensive participants (*n* = 34), missing data of MMSE score (*n* = 3945), and participants with stroke (n = 762). Finally, 9527 hypertensive participants were analyzed in this study. The screening process was detailed in Figure S1.

### 2.2. Data Collection

The researchers had received standardized training and administered the questionnaires and clinical examination at baseline following a standard operating procedure. Standard questionnaires included basic personal information (such as age, sex, and education), sleep status (such as sleep duration), medical and medication history, lifestyle (such as physical activity, smoking status, and drinking status), previous disease history (such as hypertension, coronary heart disease, diabetes, and stroke).

Self-reported sleep duration was obtained by sleep questionnaire. Self-reported sleep duration was evaluated by responding to the question: “How many hours do you usually sleep every day?,” and three response choices (<5, 5–8, and ≥8 h) were provided.

MMSE was a widely used cognitive questionnaire designed to assess the cognitive performance and quantify cognitive performance in the current study [17, 18]. The scale assesses cognitive performance based on five components: orientation force (10 points), short-term memory (3 points), attention and calculation (5 points), immediate recall (3 points), and language and praxis (9 points). Scoring was done by adding the points of each part. Thus, the total score was 30, and higher MMSE scores indicate better cognitive performance.

Anthropometric parameter indicators of the clinical examination included weight, height, and systolic and diastolic blood pressure. The body mass index (BMI) was calculated according to the formula BMI = weight/height^2^ (kg/m^2^). Participants were asked to remain in a seated position after resting for 10 min before measuring blood pressure. Electronic sphygmomanometers (Omron) were used to measure blood pressure, and the measurement was taken three times, and the mean was taken. BP control maintained was defined as SBP < 140 mmHg and DBP < 90 mmHg [19].

### 2.3. Laboratory Assays

Fasting venous blood samples were collected at the baseline and processed and analyzed at the National Clinical Research Center for Kidney Disease, Guangzhou, China. Automatic clinical analyzers (Beckman Coulter) were used to measure fasting glucose, lipids (total cholesterol, high-density lipoprotein-cholesterol (HDL-C) and low-density lipoprotein cholesterol (LDL-C), triglycerides), and homocysteine (Hcy). Estimating the glomerular filtration rate (eGFR) was calculated using the Chronic Kidney Disease Epidemiology Collaboration (CKD-EPI) formula.

### 2.4. Covariables

Continuous covariates included age (years), BMI (kg/m^2^), SBP (mmHg), DBP (mmHg) triglycerides (mmol/L), total cholesterol (mmol/L), HDL-C (mmol/L), LDL-C (mmol/L), homocysteine (*μ*mol/L), and eGFR (mL/min/1.73m^2^), and categorical covariates included sex (male, female), education (illiteracy, primary, secondary, and above), physical activity (mild, moderate, vigorous), current smoking (yes, no), current drinking (yes, no), antihypertensive medication (yes, no), coronary heart disease (yes, no), and diabetes (yes, no). Participants who had smoked within the past 30 days were defined as current smokers. Current drinking was defined as drinking at least once in the past 30 days.”

### 2.5. Statistical Analysis

Data of continuous variables and categorical variables were, respectively, presented as mean ± standard deviation (SD) and frequency (%). The comparison of the baseline characteristics across different groups by sleep duration and sex were evaluated by analysis of variance (ANOVA) tests or Chi-square tests. The linear regression models (beta coefficient (*β*) and 95% confidence interval (CI)) adjusted for the main covariates were designed to assess the independent association between sleep duration and cognitive performance. Model was adjusted for sex, age, education, physical activity, current smoking, current drinking, BMI, SBP, DBP, coronary heart disease, diabetes, antihypertensive drugs, antidiabetic drugs, statin-lowering drugs, Hcy, TG, HDL-C, LDL-C, and eGFR, except for the variable that was stratified. The adjustment of covariates is based on clinical significance, a potential confounding effect of at least 5%, and statistical significance in univariate analysis. Moreover, we conducted subgroup analyses for age, education setting, current smoking, current drinking, diabetes, LDL-C, Hcy, BMI, and BP control maintained to explore the potential factors modifying the association.

The statistical package R (http://www.r-project.org) and Empower (R) (http://www.empowerstats.com; X&Y Solutions, Inc., Boston, MA) were used to perform all analyses. A 2-tailed *P* < 0.05 was considered significant.

## 3. Results

### 3.1. Baseline Characteristics

Participants' characteristics were presented by sleep duration (<5 h, 5-8 h, and ≥8 h) and sex in Table 1. A total of 9527 subjects were included. The average age was 63.7 ± 9.8 years. The proportion of illiterate in female was far beyond that of male. In male, those who slept ≥8 hours were more likely to be older and illiterate and had lower BMI, DBP, TG, eGFR, and MMSE score compared to those who slept 5-8 hours. In female, those who slept ≥8 hours were more likely to be illiterate, had higher Hcy and fasting glucose, and lower MMSE score.

### 3.2. Association between Sleep Duration and MMSE Score

**Figure 1:**
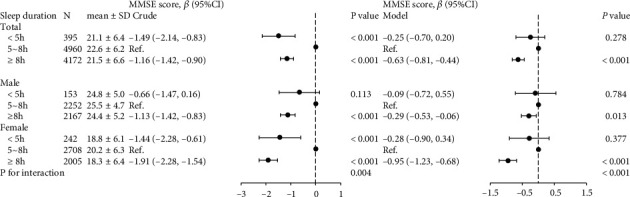
Sex differences in the association between sleep duration and cognitive performance. CI: confidence interval; MMSE: Mini-Mental State Examination; BMI: body mass index; SBP: systolic blood pressure; DBP: diastolic blood pressure; Hcy: homocysteine; TG: triglycerides; HDL-C: high-density lipoprotein cholesterol; LDL-C: low-density lipoprotein cholesterol; eGFR: estimated glomerular filtration rate. P for interaction: 2-way interaction of sleep duration and sex on cognitive performance. The multivariate model adjusted for sex, age, education, physical activity, current smoking, current drinking, BMI, SBP, DBP, coronary heart disease, diabetes, antihypertensive drugs, antidiabetic drugs, statin-lowering drugs, Hcy, TG, HDL-C, LDL-C, and eGFR, except for the variable that was stratified.

Figure 1 showed the association of sleep duration with MMSE score. In total population, compared to reference sleep duration (5-8 h), only long sleep duration (≥8 h) was independently and inversely associated with MMSE score (*β* = −0.63, 95% CI: -0.81 to -0.44, *P* < 0.001) after adjusting for main confounders. Furthermore, this study investigated sex differences in the association between sleep duration and MMSE score. We still observed the association between long sleep duration (≥8 h) and MMSE score adjusting for pertinent covariables in both male and female. However, the association between the two was stronger in female (*β* = −0.95, 95% CI: -1.23 to -0.68, *P* < 0.001) than in male (*β* = −0.29, 95% CI: -0.53 to -0.06, *P* = 0.013). Moreover, the test for interaction between sex and sleep duration on MMSE score was significant (*P* − interaction < 0.001).

Furthermore, after adjustment for main confounders, long sleep duration (≥8 h) was also inversely related to the subscores of MMSE in female and total population (all *P* < 0.05), including orientation force, short-term memory, attention and calculation, immediate recall, and language and praxis (Figure S2-6).

### 3.3. Subgroup Analyses by Potential Effect Modifiers

**Figure 2:**
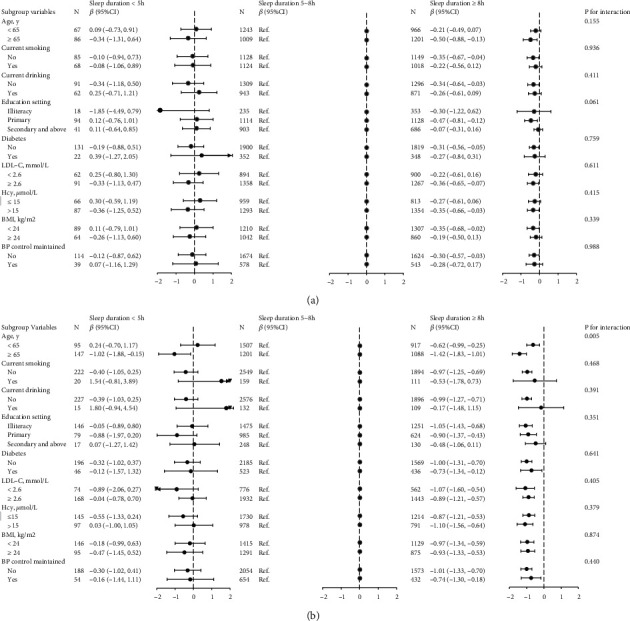
Stratified analyses of the association between sleep duration and cognitive performance by sex. (a) Male. (b) Female. Adjusted for age, education, physical activity, current smoking, current drinking, BMI, SBP, DBP, coronary heart disease, diabetes, antihypertensive drugs, antidiabetic drugs, statin-lowering drugs, Hcy, TG, HDL-C, LDL-C, and eGFR, except for the variable that was stratified. CI, confidence interval; BMI: body mass index; SBP: systolic blood pressure; DBP: diastolic blood pressure; Hcy: homocysteine; TG: triglycerides; HDL-C: high-density lipoprotein cholesterol; LDL-C: low-density lipoprotein cholesterol; eGFR: estimated glomerular filtration rate.

Stratified analyses by sex were conducted to reveal the association between sleep duration (<5 h, 5-8 h, and ≥8 h) and cognitive performance in different subgroups (Figure 2). No significant interactions were found in the subgroups, including current smoking, current drinking, education setting, diabetes, LDL-C, Hcy, BMI, and BP control maintained subgroups (all *P* ≥ 0.05), in both male and female.

However, there was a significant interaction between sleep duration and age on cognitive performance only in female. A stronger positive association between long sleep duration and worse cognitive performance was found in female aged ≥65 years (*β* = −1.42; 95% CI: -1.83 to -1.01) compared with female aged <65 years (*β* = −0.62; 95% CI: -0.99 to -0.25; *P* for interaction = 0.005).

## 4. Discussion

The study represented was conducted to investigate sex differences in the association of sleep duration with cognitive performance in the adults with hypertension. This study found that long sleep duration (≥8 h) was inversely associated to MMSE, especially in female. Furthermore, we also found that older people were a higher-risk population with lower cognition level in female.

A significant association of long sleep duration with cognitive performance has been previously reported. Blackwell et al. [20] demonstrated that self-reported long sleep duration (≥8 h) was related to worse cognition among older males living alone. Low et al. [21] revealed that longer sleep duration presented worse cognitive performance in US older adults. Kondo et al. [22] found the association between long sleep duration and cognitive impairment. These literatures supported our finding that long sleep duration (≥8 h) presented worse cognitive performance. The exact pathophysiological mechanisms of the association of longer sleep duration with worse cognitive performance were yet to be fully determined. The plausible explanation was that long sleep duration might indicate poor sleep quality [23], which suggested longer rest duration is required for people to recover from their baseline mental status.

Moreover, this study found the association of sleep duration with cognitive performance was stronger in female than in male. However, the mechanism for the effect of sex on the association between the two was unclear. A possible explanation is that estrogen had a protective effect on cognitive function. The rapidly decreased level of estrogen in postmenopause female might increase the risk of cognitive decline [24]. Although androgen was also related to brain protecting, bioavailable testosterone decreased at much slower rate than estrogen in the normal aging process [25–27]. Furthermore, in female, we observed that the association was more pronounced in the older. This may be related to the moderating effect of dopamine on cognitive performance, which progressively decreases with age, resulting in lower cognitive performance in older people compared to younger people [28]. However, sex differences in the association did not appear to be found in the general population in previous studies [29, 30]. This suggested hypertension appears to play a significant role in the sex differences in the association. As far as we know, hypertension is a risk factor for cognitive decline, and abnormal sleep duration is also a risk factor for hypertension. At baseline of this study, female had higher SBP than male, which might explain the finding that the association was more pronounced in female with hypertension. Additionally, a meta-analysis reported that the association between longer sleep duration and higher risk of hypertension was more pronounced in female, further supporting our findings [31].

However, the association of short sleep duration with cognitive performance remained controversial [32]. This might be due to the differences in assessment methods among these studies. Previous studies assessed sleep duration by self-report questionnaire, actigraph, or polysomnography. Using different cognitive questionnaires or computer-based testing to evaluate cognitive performance [20, 21, 33–35]. It resulted in the definition of our sleep duration and the level of cognitive performance evaluated by our studies varied between previous studies. This study performed questionnaires to collect self-reported sleep duration, used MMSE to assess cognitive performance, and reported that no significant association between short sleep duration and cognitive performance. Moreover, Virta et al. [33] performed an observational follow-up study to evaluate the association of self-reported sleep duration with cognitive function assessed by telephone interview. Toschi et al. [35] also collected self-reported sleep duration and cognitive function measured by questionnaires. These studies all denied the association between short sleep duration and cognitive function. These studies were consistent with the results of this study.

Of course, several limitations of this study should not be ignored. First, the sample size of short sleep duration in this study was relatively small. Second, the limitation of the MMSE questionnaire is its poor sensitivity to people with mild cognitive impairment. This study involved a large proportion of poorly educated subjects with limited understanding of the questionnaire. Therefore, the MMSE questionnaire was more suitable for large-scale epidemiological studies and daily practice due to its greater comprehensibility compared to other questionnaires and operability compared to other cognitive screening devices. Finally, females in this study were less educated than male, which may impair their understanding of the questionnaire. Thus, we adjusted for education as a confounding factor in the model to reduce this effect.

## 5. Conclusion

In summary, this study found that long sleep duration (≥8 h) was associated with poorer cognitive performance. Furthermore, this association was more pronounced in female than in male, especially in older female. The results suggested that elderly female with hypertension need to pay more attention to the management of sleep time to reduce the risk of low cognitive performance compared to male. The result needed more data from clinical practice to further verify in the future.

## Figures and Tables

**Table 1:** Baseline characteristics of study participants.

Variables	Total	Sleep duration							
		Male				Female			
		<5 h	5 ~ 8 h	≥8 h	*P* value	<5 h	5 ~ 8 h	≥8 h	*P* value
*N*	9527	153	2252	2167		242	2708	2005	
Age (y)	63.7 ± 9.8	64.3 ± 9.5	62.1 ± 10.2	64.9 ± 10.2	<0.001	66.9 ± 8.5	62.7 ± 9.1	64.9 ± 9.6	<0.001
BMI (kg/m^2^)	23.6 ± 3.5	23.4 ± 3.5	23.7 ± 3.5	23.1 ± 3.5	<0.001	23.2 ± 3.4	24.0 ± 3.6	23.6 ± 3.5	<0.001
Current smoking, *n* (%)	2500 (26.2)	68 (44.4)	1124 (49.9)	1018 (47.0)	0.092	20 (8.3)	159 (5.9)	111 (5.5)	0.232
Current drinking, *n* (%)	2132 (22.4)	62 (40.5)	943 (41.9)	871 (40.2)	0.521	15 (6.2)	132 (4.9)	109 (5.4)	0.523
SBP (mmHg)	147.2 ± 17.5	144.7 ± 16.5	144.4 ± 17.1	146.0 ± 17.7	0.007	150.2 ± 16.9	148.5 ± 17.3	149.9 ± 17.4	0.011
DBP (mmHg)	89.0 ± 10.8	90.2 ± 11.8	90.6 ± 10.7	89.8 ± 11.3	0.048	87.7 ± 10.3	88.1 ± 10.1	87.6 ± 10.7	0.194
Education setting, *n* (%)					<0.001				<0.001
Illiteracy	3478 (36.5)	18 (11.8)	235 (10.4)	353 (16.3)		146 (60.3)	1475 (54.5)	1251 (62.4)	
Primary	4024 (42.2)	94 (61.4)	1114 (49.5)	1128 (52.1)		79 (32.6)	985 (36.4)	624 (31.1)	
Secondary and above	2025 (21.3)	41 (26.8)	903 (40.1)	686 (31.7)		17 (7.0)	248 (9.2)	130 (6.5)	
Physical activity, *n* (%)					0.197				0.688
Mild	5311 (55.7)	89 (58.2)	1184 (52.6)	1212 (55.9)		145 (59.9)	1527 (56.4)	1154 (57.6)	
Moderate	2219 (23.3)	36 (23.5)	586 (26.0)	520 (24.0)		45 (18.6)	603 (22.3)	429 (21.4)	
Vigorous	1997 (21.0)	28 (18.3)	482 (21.4)	435 (20.1)		52 (21.5)	578 (21.3)	422 (21.0)	
Coronary heart disease, *n* (%)	533 (5.6)	12 (7.8)	124 (5.5)	138 (6.4)	0.298	33 (13.6)	123 (4.5)	103 (5.1)	<0.001
Diabetes, *n* (%)	1727 (18.1)	22 (14.4)	352 (15.6)	348 (16.1)	0.823	46 (19.0)	523 (19.3)	436 (21.7)	0.107
Antihypertensive drugs, *n* (%)	5798 (60.9)	86 (56.2)	1323 (58.7)	1343 (62.0)	0.054	157 (64.9)	1671 (61.7)	1218 (60.7)	0.430
Antidiabetic drugs, *n* (%)	447 (4.7)	7 (4.6)	83 (3.7)	86 (4.0)	0.793	10 (4.1)	148 (5.5)	113 (5.6)	0.624
Statin-lowering drugs, *n* (%)	258 (2.7)	5 (3.3)	53 (2.4)	51 (2.4)	0.767	6 (2.5)	74 (2.7)	69 (3.4)	0.328
Hcy (*μ*mol/L)	17.9 ± 11.1	19.0 ± 9.4	20.0 ± 14.0	20.6 ± 13.5	0.218	15.5 ± 5.3	15.4 ± 7.0	16.0 ± 8.2	0.004
Fasting glucose (mmol/L)	6.2 ± 1.6	6.1 ± 1.4	6.1 ± 1.5	6.1 ± 1.5	0.766	6.2 ± 1.4	6.2 ± 1.5	6.4 ± 1.8	0.006
TCHO (mmol/L)	5.1 ± 1.1	4.9 ± 1.0	4.9 ± 1.1	4.9 ± 1.0	0.334	5.3 ± 1.2	5.3 ± 1.1	5.3 ± 1.1	0.369
TG (mmol/L)	1.8 ± 1.3	1.6 ± 1.2	1.8 ± 1.4	1.6 ± 1.2	0.005	1.9 ± 1.4	2.0 ± 1.2	2.0 ± 1.4	0.223
LDL-C (mmol/L)	2.9 ± 0.8	2.8 ± 0.6	2.8 ± 0.8	2.8 ± 0.8	0.067	3.0 ± 0.8	3.1 ± 0.8	3.1 ± 0.8	0.424
HDL-C (mmol/L)	1.5 ± 0.4	1.5 ± 0.5	1.5 ± 0.4	1.5 ± 0.4	0.063	1.6 ± 0.4	1.5 ± 0.4	1.5 ± 0.4	0.524
eGFR (mL/(min·1.73m^2^))	86.3 ± 19.6	83.8 ± 19.5	86.6 ± 19.3	83.0 ± 20.7	<0.001	84.3 ± 19.6	89.1 ± 18.0	86.3 ± 19.9	<0.001
MMSE score	22.1 ± 6.4	24.8 ± 5.0	25.5 ± 4.7	24.4 ± 5.2	<0.001	18.8 ± 6.1	20.2 ± 6.3	18.3 ± 6.4	<0.001

Values are *N* (%), mean ± SD, *P*  values are calculated using *t*-test, *χ*^2^ test or Fisher exact test. Abbreviations: BMI: body mass index; SBP: systolic blood pressure; DBP: diastolic blood pressure; Hcy: homocysteine; TCHO: total cholesterol; TG: triglycerides; HDL-C: high-density lipoprotein cholesterol; LDL-C: low-density lipoprotein cholesterol; eGFR: estimated glomerular filtration rate; MMSE: Mini-Mental State Examination.

## Data Availability

The primary data may not be shared at this point, as the data are still part of an ongoing study.

## Supplementary Materials

Supplementary Materials Figure S1: flow chart of study population inclusion. Figure S2: sex differences in the association between sleep duration and orientation force. CI: confidence interval; BMI: body mass index; SBP: systolic blood pressure; DBP: diastolic blood pressure; Hcy: homocysteine; TG: triglycerides; HDL-C: high-density lipoprotein cholesterol; LDL-C: low-density lipoprotein cholesterol; eGFR: estimated glomerular filtration rate. P for interaction: 2-way interaction of sleep duration and sex on orientation force. The multivariate model adjusted for sex, age, education, physical activity, current smoking, current drinking, BMI, SBP, DBP, coronary heart disease, diabetes, antihypertensive drugs, antidiabetic drugs, statin-lowering drugs, Hcy, TG, HDL-C, LDL-C, and eGFR, except for the variable that was stratified. Figure S3: sex differences in the association between sleep duration and immediate recall. CI: confidence interval; BMI: body mass index; SBP: systolic blood pressure; DBP: diastolic blood pressure; Hcy: homocysteine; TG: triglycerides; HDL-C: high-density lipoprotein cholesterol; LDL-C: low-density lipoprotein cholesterol; eGFR: estimated glomerular filtration rate. P for interaction: 2-way interaction of sleep duration and sex on immediate recall. The multivariate model adjusted for sex, age, education, physical activity, current smoking, current drinking, BMI, SBP, DBP, coronary heart disease, diabetes, antihypertensive drugs, antidiabetic drugs, statin-lowering drugs, Hcy, TG, HDL-C, LDL-C, and eGFR, except for the variable that was stratified. Figure S4: sex differences in the association between sleep duration and attention and calculation. CI: confidence interval; BMI: body mass index; SBP: systolic blood pressure; DBP: diastolic blood pressure; Hcy: homocysteine; TG: triglycerides; HDL-C: high-density lipoprotein cholesterol; LDL-C: low-density lipoprotein cholesterol; eGFR: estimated glomerular filtration rate. P for interaction: 2-way interaction of sleep duration and sex on attention and calculation. The multivariate model adjusted for sex, age, education, physical activity, current smoking, current drinking, BMI, SBP, DBP, coronary heart disease, diabetes, antihypertensive drugs, antidiabetic drugs, statin-lowering drugs, Hcy, TG, HDL-C, LDL-C, and eGFR, except for the variable that was stratified. Figure S5: sex differences in the association between sleep duration and short-term memory. CI: confidence interval; BMI: body mass index; SBP: systolic blood pressure; DBP: diastolic blood pressure; Hcy: homocysteine; TG: triglycerides; HDL-C: high-density lipoprotein cholesterol; LDL-C: low-density lipoprotein cholesterol; eGFR: estimated glomerular filtration rate. P for interaction: 2-way interaction of sleep duration and sex on short-term memory. The multivariate model adjusted for sex, age, education, physical activity, current smoking, current drinking, BMI, SBP, DBP, coronary heart disease, diabetes, antihypertensive drugs, antidiabetic drugs, statin-lowering drugs, Hcy, TG, HDL-C, LDL-C, and eGFR, except for the variable that was stratified. Figure S6: Sex differences in the association between sleep duration and language and praxis. CI: confidence interval; BMI: body mass index; SBP: systolic blood pressure; DBP: diastolic blood pressure; Hcy: homocysteine; TG: triglycerides; HDL-C: high-density lipoprotein cholesterol; LDL-C: low-density lipoprotein cholesterol; eGFR: estimated glomerular filtration rate. P for interaction: 2-way interaction of sleep duration and sex on language and praxis. The multivariate model adjusted for sex, age, education, physical activity, current smoking, current drinking, BMI, SBP, DBP, coronary heart disease, diabetes, antihypertensive drugs, antidiabetic drugs, statin-lowering drugs, Hcy, TG, HDL-C, LDL-C, and eGFR, except for the variable that was stratified.
