# Supplementary material for: Sex Modified the Association between Sleep Duration and worse Cognitive Performance in Chinese Hypertensive Population: Insight from the China H-Type Hypertension Registry Study
Source: Behav Neurol. 2022 Jun 24;2022:7566033. doi: 10.1155/2022/7566033 (PMC9249484; doi:10.1155/2022/7566033)
Supplement: Supplementary Materials — Figure S1: flow chart of study population inclusion. Figure S2: sex differences in the association between sleep duration and orientation force. CI: confidence interval; BMI: body mass index; SBP: systolic blood pressure; DBP: diastolic blood pressure; Hcy: homocysteine; TG: triglycerides; HDL-C: high-density lipoprotein cholesterol; LDL-C: low-density lipoprotein cholesterol; eGFR: estimated glomerular filtration rate. P for interaction: 2-way interaction of sleep duration and sex on orientation force. The multivariate model adjusted for sex, age, education, physical activity, current smoking, current drinking, BMI, SBP, DBP, coronary heart disease, diabetes, antihypertensive drugs, antidiabetic drugs, statin-lowering drugs, Hcy, TG, HDL-C, LDL-C, and eGFR, except for the variable that was stratified. Figure S3: sex differences in the association between sleep duration and immediate recall. CI: confidence interval; BMI: body mass index; SBP: systolic blood pressure; DBP: diastolic blood pressure; Hcy: homocysteine; TG: triglycerides; HDL-C: high-density lipoprotein cholesterol; LDL-C: low-density lipoprotein cholesterol; eGFR: estimated glomerular filtration rate. P for interaction: 2-way interaction of sleep duration and sex on immediate recall. The multivariate model adjusted for sex, age, education, physical activity, current smoking, current drinking, BMI, SBP, DBP, coronary heart disease, diabetes, antihypertensive drugs, antidiabetic drugs, statin-lowering drugs, Hcy, TG, HDL-C, LDL-C, and eGFR, except for the variable that was stratified. Figure S4: sex differences in the association between sleep duration and attention and calculation. CI: confidence interval; BMI: body mass index; SBP: systolic blood pressure; DBP: diastolic blood pressure; Hcy: homocysteine; TG: triglycerides; HDL-C: high-density lipoprotein cholesterol; LDL-C: low-density lipoprotein cholesterol; eGFR: estimated glomerular filtration rate. P for interaction: 2-way interaction of sleep du [file 7566033.f1.docx]

**Supplementary Material**

10289 individuals

Excluded: individuals with stroke (n=762)

9527 patients included in the final data analysis

14234 hypertensive individuals

14268 individuals enrolled in this study

Excluded: individuals missing the data of MMSE score (n=3945)

Excluded: individuals without hypertension (n=34)

Figure S1. Flow chart of study population inclusion.

Figure S2. Sex differences in the association between sleep duration and orientation force.

CI, confidence interval; BMI, body mass index; SBP, systolic blood pressure; DBP, diastolic blood pressure; Hcy, homocysteine; TG, triglycerides; HDL-C, high-density lipoprotein cholesterol; LDL-C, low-density lipoprotein cholesterol; eGFR, estimated glomerular filtration rate.

P for interaction: 2-way interaction of sleep duration and sex on orientation force.

The multivariate model adjusted for sex, age, education, physical activity, current smoking, current drinking, BMI, SBP, DBP, coronary heart disease, diabetes, antihypertensive drugs, antidiabetic drugs, statin-lowering drugs, Hcy, TG, HDL-C, LDL-C, eGFR, except for the variable that was stratified.

Figure S3. Sex differences in the association between sleep duration and immediate recall.

CI, confidence interval; BMI, body mass index; SBP, systolic blood pressure; DBP, diastolic blood pressure; Hcy, homocysteine; TG, triglycerides; HDL-C, high-density lipoprotein cholesterol; LDL-C, low-density lipoprotein cholesterol; eGFR, estimated glomerular filtration rate.

P for interaction: 2-way interaction of sleep duration and sex on immediate recall.

The multivariate model adjusted for sex, age, education, physical activity, current smoking, current drinking, BMI, SBP, DBP, coronary heart disease, diabetes, antihypertensive drugs, antidiabetic drugs, statin-lowering drugs, Hcy, TG, HDL-C, LDL-C, eGFR, except for the variable that was stratified.

Figure S4. Sex differences in the association between sleep duration and attention and calculation.

CI, confidence interval; BMI, body mass index; SBP, systolic blood pressure; DBP, diastolic blood pressure; Hcy, homocysteine; TG, triglycerides; HDL-C, high-density lipoprotein cholesterol; LDL-C, low-density lipoprotein cholesterol; eGFR, estimated glomerular filtration rate.

P for interaction: 2-way interaction of sleep duration and sex on attention and calculation.

The multivariate model adjusted for sex, age, education, physical activity, current smoking, current drinking, BMI, SBP, DBP, coronary heart disease, diabetes, antihypertensive drugs, antidiabetic drugs, statin-lowering drugs, Hcy, TG, HDL-C, LDL-C, eGFR, except for the variable that was stratified.

Figure S5. Sex differences in the association between sleep duration and short-term memory.

CI, confidence interval; BMI, body mass index; SBP, systolic blood pressure; DBP, diastolic blood pressure; Hcy, homocysteine; TG, triglycerides; HDL-C, high-density lipoprotein cholesterol; LDL-C, low-density lipoprotein cholesterol; eGFR, estimated glomerular filtration rate.

P for interaction: 2-way interaction of sleep duration and sex on short-term memory.

The multivariate model adjusted for sex, age, education, physical activity, current smoking, current drinking, BMI, SBP, DBP, coronary heart disease, diabetes, antihypertensive drugs, antidiabetic drugs, statin-lowering drugs, Hcy, TG, HDL-C, LDL-C, eGFR, except for the variable that was stratified.

Figure S6. Sex differences in the association between sleep duration and language and praxis.

CI, confidence interval; BMI, body mass index; SBP, systolic blood pressure; DBP, diastolic blood pressure; Hcy, homocysteine; TG, triglycerides; HDL-C, high-density lipoprotein cholesterol; LDL-C, low-density lipoprotein cholesterol; eGFR, estimated glomerular filtration rate.

P for interaction: 2-way interaction of sleep duration and sex on language and praxis.

The multivariate model adjusted for sex, age, education, physical activity, current smoking, current drinking, BMI, SBP, DBP, coronary heart disease, diabetes, antihypertensive drugs, antidiabetic drugs, statin-lowering drugs, Hcy, TG, HDL-C, LDL-C, eGFR, except for the variable that was stratified.
